# Targeting HGF/c-Met Axis Decreases Circulating Regulatory T Cells Accumulation in Gastric Cancer Patients

**DOI:** 10.3390/cancers13215562

**Published:** 2021-11-05

**Authors:** Juliette Palle, Laure Hirsch, Alexandra Lapeyre-Prost, David Malka, Morgane Bourhis, Simon Pernot, Elie Marcheteau, Thibault Voron, Florence Castan, Ariane Lacotte, Nadine Benhamouda, Corinne Tanchot, Eric François, François Ghiringhelli, Christelle de la Fouchardière, Aziz Zaanan, Eric Tartour, Julien Taieb, Magali Terme

**Affiliations:** 1 Université de Paris, Inserm, PARCC, 75015 Paris, France; juliette.palle@aphp.fr (J.P.); laure.hirsch@wanadoo.fr (L.H.); lapeyrea@ipc.unicancer.fr (A.L.-P.); morgane.bourhis@inserm.fr (M.B.); s.pernot@bordeaux.unicancer.fr (S.P.); elie.marcheteau@gmail.com (E.M.); thibault.voron@aphp.fr (T.V.); arianelacotte@gmail.com (A.L.); nadine.benhamouda@aphp.fr (N.B.); corinne.tanchot@inserm.fr (C.T.); eric.tartour@aphp.fr (E.T.); 2 Department of GI Oncology, AP-HP, Hôpital Européen Georges-Pompidou, Université de Paris, 75015 Paris, France; aziz.zaanan@aphp.fr (A.Z.); julien.taieb@aphp.fr (J.T.); 3 Equipe Labellisée Ligue Contre le Cancer, 31037 Toulouse, France; 4 Department of Medical Oncology, Hopital Cochin, Assistance Publique-Hôpitaux de Paris, Université de Paris, 75015 Paris, France; 5 Département de Médecine Oncologique, Gustave Roussy, Université Paris-Saclay, 94805 Villejuif, France; david.malka@gustaveroussy.fr; 6 Biostatistics Unit, CTD INCa, ICM-Montpellier Cancer Institute, 34090 Montpellier, France; Florence.Castan@icm.unicancer.fr; 7 Department of Immunology, AP-HP, Hopital Européen Georges Pompidou, 75015 Paris, France; 8 Centre Antoine-Lacassagne, 06100 Nice, France; eric.francois@nice.unicancer.fr; 9 Centre Georges-François Leclerc, 21000 Dijon, France; fghiringhelli@cgfl.fr; 10 Medical Oncology Department, Centre Léon Bérard, 69008 Lyon, France; christelle.delafouchardiere@lyon.unicancer.fr; 11 Cancer Research Center of Lyon (CRCL), UMR INSERM 1052, CNRS 5286, 69373 Lyon, France

**Keywords:** hepatocyte growth factor, c-Met, pro-angiogenic factor, regulatory T cells, gastric cancer, targeted therapies, anti-HGF

## Abstract

**Simple Summary:**

Restoring an effective immune response is the key goal of immunotherapy. One of the major mechanisms of tumor-induced immunosuppression is regulatory T cells (Treg) accumulation. In this study, using in vitro and in vivo analysis, we assessed the impact of the HGF/c-Met pathway, involved notably in tumor angiogenesis, on Treg accumulation in patients with gastric cancer. First, we reported that c-Met is expressed on circulating monocytes of gastric cancer patients and this expression seems to be associated with the worst outcome. Secondly, during in vitro cultures, c-Met+ monocytes differentiate into dendritic cells with tolerogenic properties able to induce the proliferation of Treg. Finally, rilotumumab, an anti-HGF antibody, decreases the percentage of circulating Treg in gastric cancer patients. Using HGF/c-Met inhibitors to partially reverse immunosuppression could lead to the development of new treatment associations, for example with immune checkpoint blockers.

**Abstract:**

Elucidating mechanisms involved in tumor-induced immunosuppression is of great interest since it could help to improve cancer immunotherapy efficacy. Here we show that Hepatocyte Growth Factor (HGF), a pro-tumoral and proangiogenic factor, and its receptor c-Met are involved in regulatory T cells (Treg) accumulation in the peripheral blood of gastric cancer (GC) patients. We observed that c-Met is expressed on circulating monocytes from GC patients. The elevated expression on monocytes is associated with clinical parameters linked to an aggressive disease phenotype and correlates with a worse prognosis. Monocyte-derived dendritic cells from GC patients differentiated in the presence of HGF adopt a regulatory phenotype with a lower expression of co-stimulatory molecules, impaired maturation capacities, and an increased ability to produce interleukin-10 and to induce Treg differentiation in vitro. In the MEGA-ACCORD20-PRODIGE17 trial, GC patients received an anti-HGF antibody treatment (rilotumumab), which had been described to have an anti-angiogenic activity by decreasing proliferation of endothelial cells and tube formation. Rilotumumab decreased circulating Treg in GC patients. Thus, we identified that HGF indirectly triggers Treg accumulation via c-Met-expressing monocytes in the peripheral blood of GC patients. Our study provides arguments for potential alternative use of HGF/c-Met targeted therapies based on their immunomodulatory properties which could lead to the development of new therapeutic associations in cancer patients, for example with immune checkpoint inhibitors.

## 1. Introduction

One of the potential obstacles to reducing the efficacy of immunotherapy resides in the presence of immunosuppressive cells such as regulatory T cells (Treg). Treg are enhanced in the peripheral blood and abundantly infiltrate tumor tissues in cancer patients. They hamper an efficient tumor immunity and represent a key mechanism of tumor evasion [1]. Thus, identification of factors influencing Treg accumulation in tumor-bearing hosts is essential for the understanding of Treg biology and could provide therapeutic targets.

We have previously shown that a pro-angiogenic factor, the vascular endothelial growth factor-A (VEGF-A) which is highly produced in tumor-bearing hosts, could be involved in the accumulation of Treg and that anti-angiogenic (AA) molecules targeting the VEGF-A/VEGFR pathway could decrease Treg in a mouse model of colorectal cancer and metastatic colorectal cancer patients [2]. However, other pro-angiogenic factors could be produced by the tumor or by cells from the tumor microenvironment such as Hepatocyte Growth Factor (HGF) [3]. HGF, which is the ligand of the c-Met receptor, is involved in the regulation of different cellular properties including cell proliferation, invasion, angiogenesis, and plays a key role in physiological processes such as embryogenesis, tissue repair, and regeneration [4]. The HGF/c-Met pathway is aberrantly activated or overexpressed in epithelial cancers such as gastric, lung, or ovarian cancers [5] and is an important actionable target in many solid tumors. In two meta-analyses with more than 2000 gastric cancer (GC) patients, *c-Met* amplification or overexpression is associated with a poor prognosis [6,7]. C-Met expression is a predictor of invasive growth in GC [8]. High plasmatic HGF level also correlates with poor overall survival in GC patients [9]. Beyond these cellular properties, the HGF/c-Met pathway has also been involved in immune responses [10,11], notably by different works performed in experimental animal models of inflammatory or autoimmune diseases. In a mouse model of experimental autoimmune encephalitis, overexpression of HGF in neurons or systemic administration of HGF induce an increase of Treg and limit neuroinflammation [12,13]. In cancer, HGF is mainly produced by stromal cells from the tumor microenvironment but also by tumor cells themselves and peripheral blood mononuclear cells (PBMC) [14,15]. While HGF is associated with a poor prognosis particularly in patients with GC, the role of HGF in Treg accumulation in the context of cancer has not been documented. Based on these previous reports, we hypothesized that the HGF/c-Met pathway could contribute to the increase of Treg in cancer patients. In this study, we aimed to explore the role of the HGF/c-Met axis and the impact of an anti-HGF antibody (rilotumumab) on circulating Treg in GC patients.

## 2. Material and Methods

### 2.1. Patients

#### 2.1.1. HEGP Cohort

80 patients with histologically proven gastric or esogastric junction adenocarcinoma treated in the Gastro-intestinal Oncology unit of Georges Pompidou European Hospital (Pr. J. Taieb) were included from January 2016 to July 2020. The study was approved by the local ethics committee (Comité de Protection des Personnes d’Ile-de-France 04/09/2014). Oral and written information was provided, and each patient signed a written consent prior to enrolment. For each patient, a blood sample was collected before or throughout planned treatment. Clinical and pathological data were obtained from medical records. Patients’ characteristics at the time of blood collection are presented in Table 1. Patients with autoimmune diseases or receiving targeted therapies or monoclonal antibodies (such as trastuzumab and ramucirumab) were excluded. Survival was evaluated in 37 patients who had received a maximum of one cycle of chemotherapy, or no treatment at all, before blood sample collection.

#### 2.1.2. PRODIGE 17-ACCORD 20-MEGA Trial

The PRODIGE 17-ACCORD 20-MEGA trial is a randomized three-arm phase II study that evaluated the association of chemotherapy (FOLFOX) combined with rilotumumab (a monoclonal antibody targeting HGF) or panitumumab (a monoclonal antibody targeting EGFR) in comparison with FOLFOX alone in 162 patients with advanced GC. The trial eligibility criteria and treatments were reported previously [16]. In 123 patients, an ancillary study has been conducted to assess the evolution of circulating immune cell populations before and during treatment. The baseline characteristics of patients included in the ancillary cohort are shown in Table 2. Blood samples were collected before (day 1) and after two cycles (day 28) of treatment. Peripheral Blood Mononuclear cells (PBMCs) were isolated from peripheral blood on Ficoll–Hypaque gradients, Treg staining was performed on fresh PBMCs and analyzed by flow cytometry (see the corresponding paragraph). The assessment of circulating immune cells was planned in the initial design of the study. The final analysis presented hereby included 64 patients for whom data on circulating Treg were available both on day 1 and day 28. Specific informed consent was required for each patient. The trial was registered with the US and French health registries (NCT01443065, EudraCT No. 2009-012797-12). The study protocol has been approved by a French national ethics committee.

#### 2.1.3. Healthy Controls

Blood samples from healthy controls (HC) were obtained at Etablissement Français du Sang (European Georges Pompidou Hospital, Paris, convention N°C CPSL UNT-N°13/R/013).

### 2.2. Monocyte Isolation and Culture

PBMCs were isolated by Pancoll density gradient centrifugation (PANbiotech, Aidenbach, Deutschland). CD14^+^ monocytes were isolated by magnetic labeling followed by negative magnetic selection (Pan Monocyte Isolation kit; Miltenyi Biotec, Bergisch Gladbach, Germany). The purity of the obtained monocyte fraction was verified by immunostaining. Monocytes were then cultured at 37 °C in AIMV-Albumax medium (Thermofisher Scientific, Walltam, Massachusetts, USA) at the concentration of 1 × 10^6^ cells/mL, supplemented with human Granulocyte-Macrophage Colony-Stimulating Factor (GM-CSF) (1000 UI/mL; Miltenyi Biotec, Bergisch Gladbach, Germany) and human Interleukin 4 (IL-4) (400 UI/mL; Miltenyi Biotec) (=control condition) or GM-CSF, IL-4 and human HGF (=HGF condition) (20 ng/mL; Miltenyi Biotec). On day 3, the medium was changed, and fresh cytokines were added at the same concentrations. On day 6, culture supernatants and obtained cells were collected. Cytokine dosage and cellular immunostaining were then performed (see corresponding paragraphs). In 7 independent experiments, to provide a maturation signal to DCs, Lipopolysaccharide (LPS) (1 μg/mL; Sigma–Aldrich, Saint-Louis, MI, USA) or control PBS was added to the culture. After 24 h, culture supernatants and cells were collected.

### 2.3. Isolation of CD4^+^ T Cells

PBMCs from HC were isolated by Pancoll density gradient centrifugation. CD4^+^ cells were then isolated by magnetic labeling followed by negative magnetic selection (Miltenyi Biotech). The purity of the obtained CD4^+^ cellular fraction was assessed by immunostaining.

### 2.4. Monocyte/Lymphocyte Co-Culture

After 6 days of culture in standard or HGF condition, monocyte-derived DCs generated from GC patients were collected as previously described. DCs were then co-cultured with freshly isolated CD4+ T cells at a ratio of 5 lymphocytes for 1 dendritic cell in AIMV-Albumax medium (Thermofisher, Waltham, MA, USA) at 37 °C for 7 days. After 7 days of co-culture, cells were collected and immunostaining was performed.

### 2.5. Immunostaining

Cells were first incubated for 15 min with Human True Stain FcX Blocking reagent (Biolegend, San Diego, California, USA) to prevent unspecific binding of the antibodies to Fc receptors. Then cells were incubated for 20 min at 4 °C with the following fluorochrome-conjugated antibodies or their corresponding isotype controls: CD14 (clone: 61D3); HLA-DR (clone: LN3); CD3 (clone: OKT3); CD56 (clone: MEM-188); CD19 (clone: HIB19); CD80 (clone: 2D10.4); CD83 (clone: HB15e); CD86 (clone: IT2.2); CD25 (clone: BC96); CD127 (clone: eBioRD); FoxP3 (clone: PCH101) (all purchased at ThermoFisher Scientific, Waltham, MA, USA); CD11c (clone: 3.9); CD16 (clone: 3G8); HLA-DR (clone: LN3); PD-L1 (clone: 29E2A3); ILT-3 (clone: ZM4.1); Tim3 (clone: F38-2E2); CD1a (clone: HI149); CD14 (clone: HCD14); CD209 (clone: 9E9A8); CD40 (clone: HB14); CD64 (clone: 10.1); CD1c (clone: L161); CD4 (clone: OKT4) (all purchased at Biolegend, USA); Ki-67 (clone: MOPC-21) purchased at BD Pharmingen (Franklin Lakes, NJ, USA); cMet APC (clone: 95106) purchased at R&D Systems (Minneapolis, MN, USA). Intracellular staining was performed using Foxp3/transcription factor staining buffer set (ThermoFisher Scientific) according to manufacturer’s recommendations. Cell viability was assessed using the LIVE/DEAD fixable dead stain kit (ThermoFisher Scientific). Samples were run through a LSRII flow cytometer or Fortessa ×20 (BD Biosciences, Franklin Lakes, NJ, USA) using DIVA^®^ software (BD Biosciences, Franklin Lakes, New Jersey, USA). Analysis were performed using FlowJo^®^ software (Flow Jo, Ashland, OR, USA). Dead cells and doublet cells were excluded from the analysis. Isotype controls and fluorescence minus one controls were used as negative controls.

### 2.6. Analysis of Cytokine Production

IL-10 and TGF-β1 levels in culture supernatants were assessed by enzyme-linked immunoabsorbent assays (ELISAs) using commercially available kits (respectively purchased at Biolegend and R&D Systems). The limits of detection were 3.9 pg/mL for IL-10 and 31.2 pg/mL for TGF-β1. HGF and IL-10 levels in plasma from GC patients and HC were assessed by enzyme-linked immunoabsorbent assays (ELISAs) using a commercially available kit (respectively R&D Systems and Biolegend). The limit of detection for HGF was 156 pg/mL.

### 2.7. Statistical Analyses

Results are presented as mean and Standard Error of the Mean (SEM). All statistical analyses were performed with GraphPad Prism^®^ software (GraphPad Software, San Diego, CA, USA). Comparisons were performed with Wilcoxon, Mann Whitney, or Kruskal Wallis test, as appropriate. Overall survival (OS) was defined as the time from randomization to death by any cause. OS was estimated by the Kaplan-Meier method. Univariate analysis was performed using a proportional hazard model to estimate the hazard ratio. Comparisons were performed using the log-rank test. A *p*-value < 0.05 was considered statistically significant. All statistical tests were two-sided.

## 3. Results

### 3.1. C-Met Receptor Is Expressed on Peripheral Blood Monocytes in GC Patients

**Figure 1:**
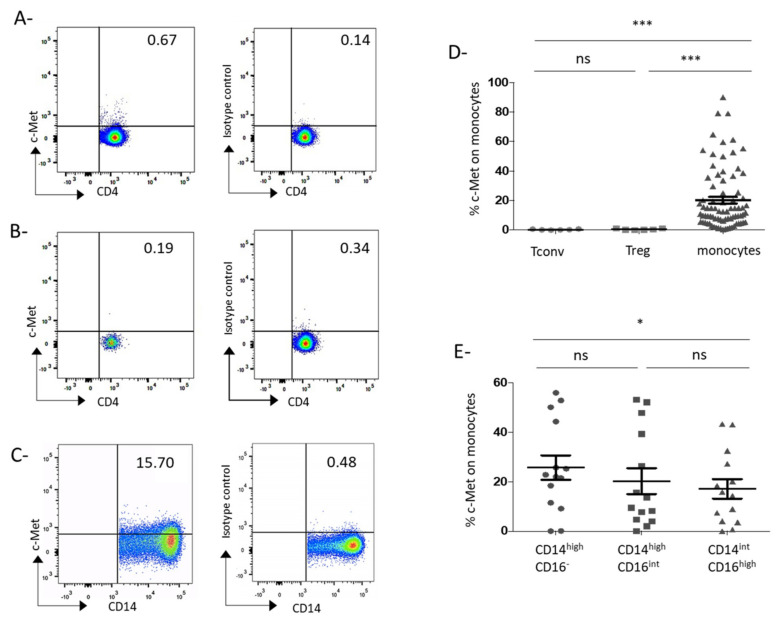
Circulating monocytes but not circulating conventional or regulatory T cells express the c-Met receptor in GC patients. Freshly isolated PBMCs from GC patients were stained with fluorochrome-conjugated antibodies or their isotype control and analyzed by flow cytometry. Tconv were defined as CD4^+^/CD25^−^ cells. Treg were defined as CD4^+^/CD25^+^/Foxp3^+^/CD127^low^cells. Monocytes were defined as CD14^+^/HLA-DR^+^/CD3^−^/CD56^−^/CD19^−^ cells. (**A**–**C**) Representative c-Met expression by Tconv (**A**), Treg (**B**), and monocytes (**C**) of GC patients. Results are indicated in percentages of c-Met positive cells. (**D**) Mean c-Met expression by peripheral Tconv (mean ± SEM: 0.36 ± 0.13%; *n* = 6), Treg (mean ± SEM: 0.55 ± 0.20%; *n* = 6) and monocytes (mean ± SEM: 20.24 ± 2.32%; *n* = 80) from GC patients. *** indicates a *p*-value ≤ 0.001 according the Kruskall-Wallis multiple comparisons test. (**E**) Mean c-Met expression by the three monocytes subpopulations from GC patients (*n* = 14): CD14^high^/CD16^−^ (mean ± SEM: 25.67 ± 4.95%), CD14^high^CD16^int^ (mean ± SEM: 20.24 ± 5.26%) and CD14^int^CD16^high^ (mean ± SD: 17.10 ± 3.914%). * indicates a *p*-value ≤ 0.05 according the Friedman multiple comparisons test. Ns: nonsignificant.

**Figure 2:**
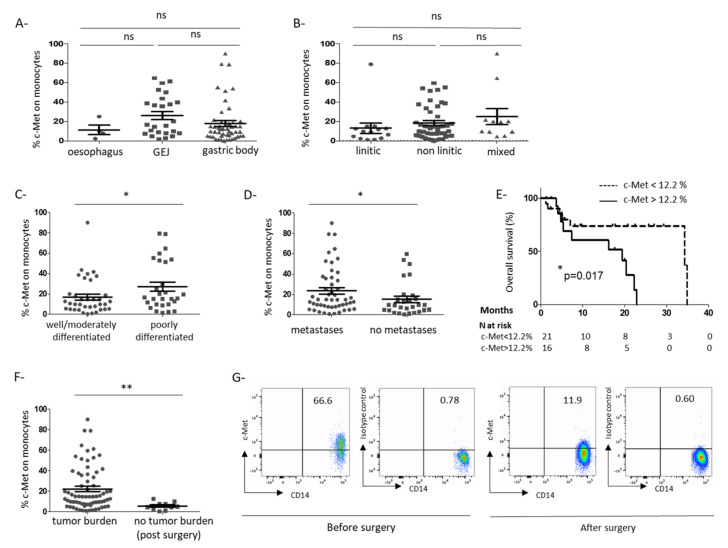
The C-Met expression on circulating monocytes is associated with an aggressive disease phenotype in GC patients. Clinical and pathological characteristics of the GC patients included in our cohort were analyzed (*n* = 80) (**A**–**D**,**F**). C-Met expression was assessed by flow cytometry as in Figure 1. Mean c-Met expression on circulating monocytes according to tumor location (esophagus, gastro–esophagic junction (GEJ), gastric body) (**A**), linitic status (**B**), tumor differentiation (**C**), presence of metastases (**D,**) and presence of an evolutive tumor burden (**F**). * indicates a *p*-value ≤ 0.05 and ** indicates a *p*-value ≤ 0.01 according to the Mann-Whitney test. NS: nonsignificant (**E**) For 37 patients who had received 1 or 0 cycles of chemotherapy before assessment of c-Met expression, survival probability has been estimated according to c-Met expression level on monocytes (high expression > median *n* = 16 (full line), lower expression < median, *n* = 21 (dashed line)). The median was calculated on the global population. (*p* = 0.017 according the Log-rank test). (**G**) For one patient with a localized tumor, the c-Met expression on circulating monocytes was assessed by flow cytometry before and after tumor resection (gastrectomy). Results are indicated as percentages of c-Met positive cells.

A prospective cohort of GC patients has been set up. Patients’ characteristics are reported in Table 1. Plasmatic HGF level was increased in GC patients compared to HC patients (1407 ± 126 pg/mL vs. 658.6 ± 153 pg/mL, *p* = 0.031; Supplementary Figure S1A) and was associated with a more advanced disease phenotype since metastatic patients exhibited higher levels of plasmatic HGF than patients with localized disease (1726 ± 136.3 pg/mL vs. 1010 ± 152.9 pg/mL, *p* = 0.0047; Supplementary Figure S1B), as previously reported [17]. We first analyzed the expression of c-Met in the peripheral blood of GC patients from our cohort. A marginal expression of c-Met was found on Treg and conventional T cells (0.55 ± 0.20% on Treg and 0.36 ± 0.13% on Tconv, Figure 1A,B,D) but c-Met was expressed on circulating monocytes (20.24 ± 2.32%) (Figure 1C,D). In 14 patients, we assessed c-Met on different monocyte subsets characterized by the expression of CD14 and CD16 [18]. C-Met was present on all monocyte subsets, however its expression level seemed to be lower in CD14^int^/CD16^hi^ subset in comparison to CD14^high^/CD16^−^ population (Figure 1E) (respectively 17.10 ± 3.91% and 25.67 ± 4.95%, *p* = 0.0166). As observed in Figure 1D, the c-Met expression on monocytes is heterogeneous among the patients and distributed between 0.38 and 90%. We then sought an association between c-Met expression on monocytes and clinical features. The c-Met expression did not vary according to tumor location (Figure 2A). Histological features such as tumor differentiation or linitic phenotype (including diffuse carcinomas according to Lauren’s classification and signet ring cell carcinomas according to OMS’ classification [19]) are of importance since they are associated with different clinical outcomes and different immune infiltrate as we previously showed [20]. No correlation has been observed between linitic phenotype and c-Met expression (Figure 2B) but a poor differentiation grade was associated with an enhanced c-Met expression on monocytes compared to well or moderately differentiated tumors (27.1 ± 4.6% vs. 17.1 ± 2.9%; *p* = 0.046) (Figure 2C). Interestingly, the presence of metastases was linked to a higher c-Met expression on monocytes (metastatic: 23.6 ± 3.2% vs. localized or locally advanced: 15.3 ± 3.2%; *p* = 0.043) (Figure 2D). Thus, it seemed that c-Met expression on circulating monocytes was associated with an aggressive disease phenotype. To further assess the prognostic significance of c-Met expression by peripheral monocytes, we compared the survival probability of patients harboring a lower c-Met expression on monocytes (inferior to the observed median value on the global population = 12.2%) with those harboring a higher c-Met expression (above median value) before any treatment or after no more than one cycle of treatment (survival population), using a univariate model. The characteristics of the survival population are presented in Supplemental Table S1. In our survival population, a higher c-Met expression on circulating monocytes was associated with poorer survival (median OS: 19.4 vs. 34.9 months) (*p* = 0.017) (Figure 2E).

Furthermore, the c-Met expression on monocytes was decreased in patients who underwent tumor resection by gastrectomy (patients with a resected localized tumor) compared to patients with an active tumor burden (patients with a non-resected localized tumor or metastatic patients) (5.62 ± 1.24% and 22.33 ± 2.55%, respectively; *p* = 0.001) (Figure 2F). These results suggested that the presence of the tumor is involved in the c-Met expression on monocytes in GC patients. For one patient, we could obtain peripheral blood before and after tumor resection by gastrectomy. In this patient, c-Met was highly expressed on monocytes before surgery (66.6%) and dropped after resection of the tumor (11.9%; Figure 2G). In parallel, HGF plasmatic level was significantly enhanced in the plasma of patients with more than 5% of monocytes expressing c-Met (Supplementary Figure S1C).

Thus, c-Met is expressed on circulating monocytes and is associated with poor tumor differentiation, the presence of metastases, and poorer survival in GC patients. Furthermore, high c-Met expression is linked to the presence of a tumor burden suggesting that a tumor-derived factor could be involved in the c-Met expression on monocytes.

### 3.2. HGF Induces the Differentiation of Monocytes into Dendritic Cells with Tolerogenic Properties

**Figure 3:**
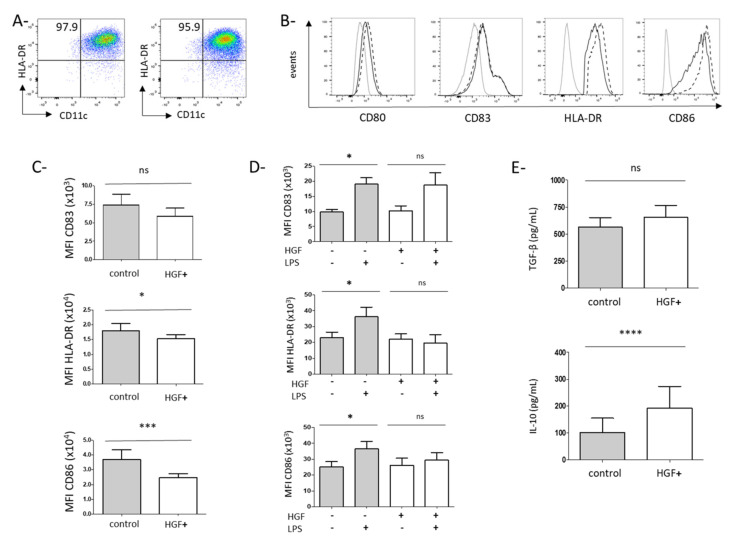
HGF modulates monocyte-derived DC differentiation from GC patients into a regulatory phenotype in vitro. Freshly isolated peripheral monocytes from GC patients were cultured in the presence of GM-CSF and IL-4 with HGF (=HGF condition) or without HGF (=control condition) as described in the material and method section. After 6 days, cell phenotype was assessed by flow cytometry. In both conditions, monocyte-derived cells harbored DC features such as CD11c and HLA-DR co-expression. (**A**) CD11c and HLA-DR expression by monocyte-derived DCs without (left panel) or with HGF (right panel). Results are indicated as percentages of CD11 HLA-DR^+^ cells. Representative staining out of 45 independent experiments with similar results is shown. (**B**) Expression of CD80, CD83, HLA-DR, and CD86 in DCs generated without (black dashed curve) or with HGF (black full curve). Markers were set on their proper isotype control (light grey curve). A representative staining out of respectively 23, 45 and 29 independent experiments is shown (**C**) Median Fluorescence Intensity (MFI) of CD83 (*n* = 23), HLA-DR (*n* = 45) and CD86 (*n* = 29) by DCs generated in control condition or in HGF condition. * indicates a *p*-value ≤ 0.05 according to the Wilcoxon test. *** indicates a *p*-value ≤ 0.001 according to the Wilcoxon test. NS: non significant. After 6 days of culture, DCs were maturated with LPS 1 μg/mL or control PBS. After 24 h, cells were collected, and their phenotype was studied by flow cytometry. (**D**) Mean expression (MFI) of CD83 (upper panel, *n* = 6), HLA-DR (center panel, *n* = 6) and CD86 (lower panel, *n* = 6) by DCs generated in control or HGF condition with or without LPS. * indicates a *p*-value ≤ 0.05 according to the Wilcoxon test. Culture supernatants were collected and cytokine levels were assessed by ELISA. (**E**) TGF-β levels (upper panel, *n* = 18) and IL-10 levels (lower panel, *n* = 26) in supernatants of control or HGF-generated DCs. **** indicates a *p*-value ≤ 0.0001 according to the Wilcoxon test.

We next explored the impact of HGF on monocyte differentiation into DCs. Isolated CD14^+^ monocytes from peripheral blood of GC patients were cultured in a DC differentiation medium with or without HGF. After 6 days of culture, DCs were generated expressing CD11c, HLA-DR, CD209, and CD1c (Figure 3A and Figure S2). The percentage of CD11c^+^ HLA-DR^+^ cells was similar in these two conditions (86.6 ± 2.22% and 83.99 ± 2.56%, respectively) (Figure 3A). Then we analyzed the expression of costimulatory molecules on DCs cultured with or without HGF at day 6. No significant difference was observed for CD80 or CD83, but CD86 expression was significantly decreased in the presence of HGF (Median Fluorescence Intensity-MFI: 2.5 ± 0.28 × 10^4^ with HGF vs. 3.7 ± 0.65 × 10^4^ without HGF; *p* = 0.0003) (Figure 3B,C). Interestingly, CD86 was not decreased when DC was cultured in the presence of Epithelial Growth Factor (EGF), another growth factor involved in cancer development (data not shown). HLA-DR was also lowered in the presence of HGF (MFI: 1.5 ± 0.14 × 10^4^ with HGF vs. 1.8 ± 0.2 × 10^4^ without HGF; *p* = 0.033) (Figure 3B,C). HGF did not affect CD40 expression nor the expression of inhibitory receptors such as PD-L1, Tim-3, or ILT-3 (Supplementary Figure S2). To determine if HGF can impact the maturation of DCs, we stimulated DCs with LPS to induce their maturation and analyzed the expression of these different costimulatory molecules 24 h later. Whereas LPS induced upregulation of CD83, CD86, and HLA-DR on DCs generated without HGF, these maturation markers were not upregulated on DCs cultured in the presence of HGF (Figure 3D). We next evaluated immunosuppressive cytokines secretion by these DCs cultured in the presence or absence of HGF. TGF-β was detected in DCs supernatants (Figure 3E) but with no significant difference between these two culture conditions. HGF-DCs produced higher amount of IL-10 compared with DCs cultured without HGF (193.3 ± 80.32 pg/mL with HGF vs. 101.6 ± 54.16 pg/mL without HGF; *p* < 0.0001) (Figure 3E).

Thus, HGF does not prevent DCs differentiation but restrains HLA-DR and CD86 expression and full maturation induced by a maturating agent such as LPS. Furthermore, HGF enhances the production of IL-10, an immunosuppressive cytokine by DCs.

### 3.3. HGF-Generated DCs Induce the Development of Regulatory T Cells in Gastric Cancer Patients

**Figure 4:**
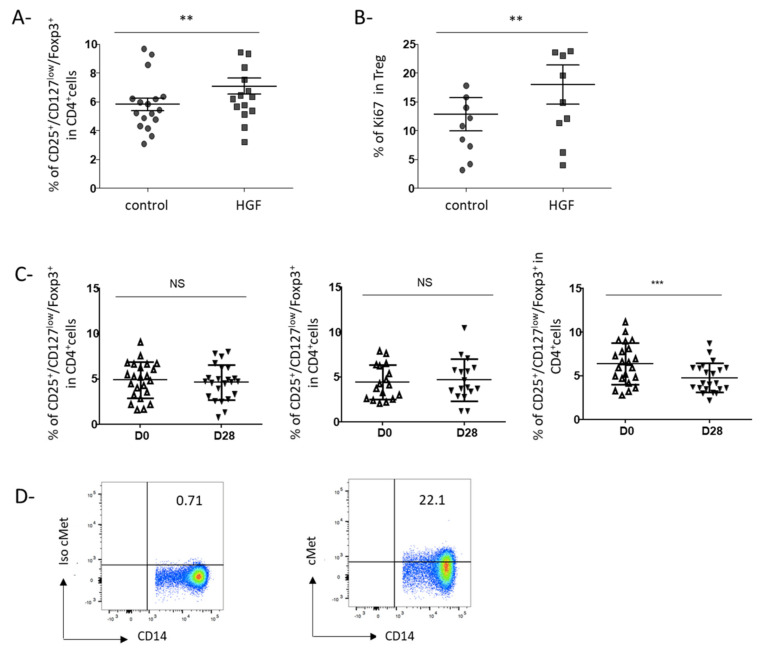
The proportion of regulatory T cells is enhanced by HGF-DCs in vitro and is decreased by rilotumumab (a monoclonal antibody targeting HGF) in vivo in gastric cancer patients. (**A**,**B**) After 6 days of culture, HGF-DCs or control-DCs were then co-cultured with freshly isolated CD4+ T cells from HC. After 7 days of co-culture, cells were collected and their phenotype was assessed by immunostaining and flow cytometry. Treg were defined as CD4^+^ CD25^+^ Foxp3^+^ CD127^low^ cells. Treg proliferation was assessed by Ki-67 intra-cellular expression. (**A**) The proportion of Treg amongst CD4^+^ T cells after 7 days of co-culture with control-DCs or HGF-DCs is shown (*n* = 17). (**B**) Ki67 was assessed in Treg cells after 7 days of co-culture with control-DCs or HGF-DCs (*n* = 10). ** indicates a *p*-value ≤ 0.01 according to the Wilcoxon test. (**C**,**D**) The PRODIGE 17-ACCORD 20-MEGA trial is a randomized three-arm phase II study that evaluated the efficacy of 3 treatment regimens in patients with advanced GC: chemotherapy (FOLFOX) alone, an association of FOLFOX + panitumumab (an anti-EGFR monoclonal antibody), or association of FOLFOX + rilotumumab (an anti-HGF monoclonal antibody). PBMCs were collected before (day 1) and after 2 cycles of treatment (day 28). Immunostaining was then performed and cells were analyzed by flow cytometry. Treg was defined as CD4^+^ CD25^+^ Foxp3^+^ CD127^low^ cells, monocytes were defined as CD3^−^/CD56^−^/CD19^−^/CD14^+^/HLA-DR^+^ cells as previously described. (**C**) Proportions of Treg amongst circulating CD4^+^ cells before and after two cycles of treatment in GC patients treated with FOLFOX (*n* = 23, left panel), FOLFOX + anti EGFR (*n* = 17, middle panel) or FOLFOX + anti HGF (*n* = 21, right panel) are shown. *** indicates a *p*-value ≤ 0.005 according to the Wilcoxon test. (**D**) Monocytes from PBMC of 9 GC patients included in the PRODIGE17-ACCORD20-MEGA trial were stained as described in Figure 1 (*n* = 9). Representative staining out of 9 independent experiments is shown. Results are indicated as percentages of c-Met positive cells.

Given the higher production of IL-10 by HGF-generated DCs and their impaired maturation, we hypothesized that HGF-DCs could be involved in Treg accumulation. To test this hypothesis, we cocultured DCs differentiated from circulating monocytes of GC patients in the presence or absence of HGF with allogeneic purified CD4^+^ T cells. HGF-generated DCs enhanced the proportion of CD25^+^Foxp3^+^CD127^lo^ Treg in coculture compared to control-DCs (*p* = 0.008) (Figure 4A). Furthermore, we observed an increase of Ki67 expression in these Treg, suggesting that HGF-generated DCs induced the proliferation of Treg in culture (*p* = 0.002) (Figure 4B).

### 3.4. Rilotumumab, an Anti-HGF Antibody, Reduces Treg Proportion in the Peripheral Blood of Advanced GC Patients

Next, we took advantage of a clinical trial (Prodige 17-ACCORD20-MEGA) evaluating the impact of an anti-HGF antibody (rilotumumab) in association with chemotherapy in advanced GC patients. In this three-arm trial, patients were randomized to receive either chemotherapy alone (Folfox), chemotherapy associated with rilotumumab, or chemotherapy associated with an anti-EGFR (panitumumab) (Table 2). We evaluated the percentage of circulating Treg before and after 2 cycles of treatment. Only patients receiving the association of chemotherapy and anti-HGF exhibited a decrease of Treg proportion after 2 cycles of treatment compared to baseline (*p* < 0.0001). On the opposite, no modification of circulating Treg proportion was observed in patients receiving chemotherapy alone or chemotherapy associated with anti-EGFR suggesting that Treg decrease was due to rilotumumab (Figure 4C). Furthermore, for 9 patients from the rilotumumab group, we had enough cells before treatment to analyze c-Met expression on monocytes. We could confirm that circulating monocytes express c-Met in this group (Figure 4D).

Thus, inhibition of HGF in advanced GC patients decreased circulating Treg.

Taken together, our findings suggest that the HGF/c-Met pathway is involved in Treg accumulation in the context of cancer. In advanced GC patients, circulating monocytes express the c-Met receptor and could differentiate into DCs with tolerogenic properties in vitro in the presence of HGF. Inhibition of the HGF/c-Met pathway could prevent Treg increase in cancer patients.

## 4. Discussion

A link between HGF and Treg has been firstly reported in mouse models of transplantation, autoimmune or inflammatory disease [10,11,12,13]. In a swine model of allogeneic kidney transplantation, administration of HGF in the graft inhibited acute renal rejection and enhanced Treg [21]. In mice, HGF overexpression in neurons increased Treg percentage in the central nervous system and inhibited the development of experimental autoimmune encephalitis. This effect was related to the development of tolerogenic DCs [12]. Furthermore, in vitro human studies have reported that HGF induced the differentiation of monocytes from healthy volunteers into DCs with tolerogenic properties [22,23]. The HGF/c-Met pathway is upregulated in many different cancer types but the influence of this pathway on Treg development in cancer has to our knowledge not been explored. In this work, we first observed that circulating CD4^+^ T cells do not express c-Met on their cell surface. This is in accordance with previous reports showing no expression of c-Met on naïve T cells, or on circulating CD4^+^ T cells from multiple sclerosis patients [24]. Only subsets of highly autoreactive encephalitogenic CD8^+^ T cells and of CD4^+^ memory T cells which preferentially recirculate in the heart have been reported to express c-Met in mice [25,26]. On the other hand, we found that c-Met was present on circulating monocytes from GC patients. In healthy volunteers, circulating monocytes have previously been shown to express low levels of c-Met which can be enhanced during activation by endotoxin [27]. C-Met was also described on circulating monocytes from multiple sclerosis patients [24]. In hepatocellular carcinoma, Zhao et al. observed a subset of peritumoral monocytes expressing c-Met [28]. As far as we know, our study is the first to show an expression of c-Met on monocytes in the peripheral blood of cancer patients. This expression is not restricted to GC since we also observed the presence of c-Met on circulating monocytes from metastatic colorectal cancer patients (*n* = 35; mean: 12.68 ± 2.90%, our unpublished data).

Our study pointed out that c-Met expression on monocytes was associated with the presence of a tumor since patients after tumor resection exhibit a lower c-Met level on monocytes compared to patients with a tumor burden (patients with a non-resected localized tumor or metastatic patients) (Figure 2F). Furthermore, in one patient we observed a decrease of c-Met after tumor resection. These results suggested that c-Met on monocytes could be induced or upregulated by a tumor-derived factor. Different works have shown that in vitro stimulation of tumor cell lines by soluble factors such as IL-1β, TNFα, IL-6 or TGFβ can enhance c-Met expression [29]. Since these cytokines are produced by tumors or by cells from the tumor microenvironment, they could also be involved in the c-Met expression on circulating monocytes from GC patients. On the other hand, HGF is also able to increase c-Met expression by an autocrine and paracrine manner on epithelial cells and tumor cell lines in vitro [30]. In this sense, we noticed that patients exhibiting an expression of c-Met above 5% have a plasmatic HGF level higher than patients with less than 5% of c-Met (Supplementary Figure S1). However, in preliminary experiments, we were not able to detect any modification of c-Met expression after HGF stimulation of monocytes (our unpublished data). Further investigations are needed to determine the mechanism of upregulation of c-Met on monocytes in GC patients.

In a study performed in 102 colorectal cancer patients, c-Met expression by the tumor was correlated to TNM status, lymph node, and liver metastases [31]. In our cohort of GC patients, the c-Met expression on monocytes was also associated with the presence of metastasis (Figure 2D). However, patients with liver metastasis did not exhibit an elevated level of c-Met expression compared to patients with other metastasis locations (data not shown). In two meta-analyses, c-Met amplification or overexpression by the tumor was related to a poor prognosis [6,7]. In our cohort, we observed that c-Met expression on monocytes was associated with more aggressive disease and poorer survival in GC patients in a univariate model (Figure 2C–E). Due to a small number of patients in our survival cohort, we were not able to perform a multivariate analysis.

Few works have previously reported the influence of HGF on monocytes in vitro. Stimulation of monocytes from HC with HGF induced the development of cells with DC features able to produce IL-10 and to generate the differentiation of Treg from allogeneic CD4^+^ T cells [23,32]. Molnarfi et al. have shown that culture of monocytes from HC in DC differentiating medium in the presence of HGF leads to the development of DC exhibiting less costimulatory molecules and HLA-DR. In this condition, HGF enhanced the capacity of DC to stimulate Treg differentiation [22]. However, no study has been performed on monocytes from cancer patients. Since we observed that in GC patients, monocytes express c-Met, we purified circulating monocytes from these patients and cultured them in a DC differentiation medium with or without HGF. In accordance with the results previously published on monocytes from HC, we observed that HGF induced the development of DC: (1) expressing less CD86 and HLA-DR molecules; (2) impaired in their maturating capacity, (3) producing the anti-inflammatory IL-10 cytokine, and (4) facilitating the accumulation of Treg from allogeneic CD4^+^ T cells (Figure 3). Interestingly, in our cohort of patients, the plasmatic level of IL-10 was correlated with the c-Met expression on monocytes (Supplementary Figure S3), suggesting that this subset could be involved in IL-10 production in cancer patients. Treg accumulation in cancer could occur through different mechanisms such as an expansion of pre-existing Treg, conversion of conventional CD4^+^ T cells into Treg [33]. Treg accumulation induced by HGF-generated DC was at least partly due to enhanced proliferation of pre-existing Treg since upregulation of Ki67, a proliferation marker, was observed. Based on our in vitro results, we hypothesized that targeting HGF could modulate Treg in tumor-bearing patients. We analyzed peripheral Treg in GC patients and observed that targeting HGF with rilotumumab decreased Treg proportion in peripheral blood (Figure 4C).

HGF/c-Met pathway has been firstly described as a proangiogenic factor since it exhibits a very potent mitogenic activity on endothelial cells. HGF promotes endothelial motogenesis, tube and capillary blood vessels formation [34]. Early studies of the proangiogenic actions of HGF attributed the effects of HGF to the induction of VEGF-A [35]. However, HGF can also stimulate angiogenesis independently of VEGF-A by inducing different signaling pathways [36]. Furthermore, HGF represents an alternative proangiogenic pathway involved in resistance to AA treatments directed against VEGF-A [37]. Targeting the HGF/c-Met pathway, especially using rilotumumab, impacts angiogenesis as evidenced by a decrease in the proliferation of endothelial cells and tube formation in pancreatic cancer [38] and glioma [39]. Assessing the impact of rilotumumab on tumor vessels in our cohort of GC patients would have been of interest but we could not get tumor specimens before and after treatment since tumors were not resected or sampled in these metastatic GC patients after rilotumumab treatment. Our work provides a novel argument supporting the involvement of tumor-derived growth factors firstly described for their protumoral and/or proangiogenic capacities, in the immune escape observed in cancer. We and others have previously shown that VEGF-A, a proangiogenic factor, has an immunomodulatory role since it can elicit the accumulation of Treg and myeloid-derived suppressor cells, inhibit the maturation of dendritic cells and induce the expression of immune checkpoints on CD8^+^ T cells in tumor-bearing hosts [2,40,41]. In this work, we found that HGF, a protumoral and proangiogenic factor, is involved in Treg increase in the peripheral blood of cancer patients. Thus, targeting the HGF/c-Met axis has an immunomodulatory impact. Results from recent phase III studies assessing HGF/c-Met targeting therapies in GC have been disappointing [16]. However, based on these immunomodulatory properties, we could consider alternative uses of HGF/c-Met inhibitors which could be associated with immunotherapies such as the anti-PD-(L)1 antibody to unleash the immune system. Notably, cabozantinib, a multikinase inhibitor, targets not only HGF/c-Met but also the VEGF/VEGFR2 pathway [42], both involved in Treg accumulation in the context of cancer. Different works have recently shown that cabozantinib can decrease peripheral Treg in the MC38-CEA colorectal cancer model in mice [43] and also in patients with platinum-refractory metastatic urothelial carcinoma [44]. Considering our results, Treg drop could be attributable to c-Met/HGF and/or VEGF/VEGFR inhibition in this context. Associations of cabozantinib with different immunotherapies are being evaluated in different clinical trials especially in GC patients (NCT04164979, NCT03539822). Recently, an association of cabozantinib with nivolumab has been reported to enhance OS and PFS in advanced renal cancer patients [45] and has been approved as first-line treatment in advanced renal cancer patients [46].

## 5. Conclusions

In conclusion, we show that c-Met is expressed on circulating monocytes from GC patients and that this expression is linked to an aggressive disease (poor differentiation, metastatic disease) and poorer survival. HGF influences the development of DC with tolerogenic properties able to induce the development of Treg. Finally, inhibition of HGF by rilotumumab inhibits the accumulation of Treg in GC patients. Using HGF/c-Met inhibition for their impact on immunosuppression could lead to the development of new therapeutic associations for example with immune checkpoint blockers.

## Figures and Tables

**Table 1:** Characteristics of patients from the HEGP cohort.

*N* = 80	*N* (%)
**Age (years)**	
median	65
**Sex**	
male	56 (70)
female	24 (30)
**Localization**	
lower esophagus	4 (5)
esogastric junction	17 (21.5)
gastric body	57 (71)
missing data	2 (2.5)
**Differentiation**	
well	8 (10)
moderate	28 (35)
poor	28 (35)
missing data	16 (20)
**Linitis**	
yes	14 (17.5)
no	45 (56)
mixed	11 (14)
missing data	10 (12.5)
**Disease status**	
local/locally advanced	28 (35)
metastatic	52 (65)
**Tumor burden**	
yes	70 (87.5)
no (post-surgery patients)	10 (12.5)
**HER2 status**	
positive	11 (13.75)
negative	61 (76.25)
missing data	8 (10)

**Table 2:** Characteristics of patients from the PRODIGE17-ACCORD20-MEGA ancillary cohort.

*N* = 80	*N* (%)
**Age (years)**	
median	65
**Sex**	
male	56 (70)
female	24 (30)
**Localization**	
lower esophagus	4 (5)
esogastric junction	17 (21.5)
gastric body	57 (71)
missing data	2 (2.5)
**Differentiation**	
well	8 (10)
moderate	28 (35)
poor	28 (35)
missing data	16 (20)
**Linitis/Diffuse type (Lauren’s classification)**	
yes	14 (17.5)
no	45 (56)
mixed	11 (14)
missing data	10 (12.5)
**Disease status**	
local/locally advanced	28 (35)
metastatic	52 (65)
**Tumor burden**	
yes	70 (87.5)
no (post-surgery patients)	10 (12.5)
**HER2 status**	
positive	11 (13.75)
negative	61 (76.25)
missing data	8 (10)
**chemotherapy received by metastatic patients**	
FOLFOX	25 (48)
FOLFIRI	6 (11.5)
FOLFOX HERCEPTIN	3 (5.8)
OTHER	8 (15.4)
NONE	7 (13.5)

## Data Availability

The data presented in this study are available on request from the corresponding author.

## Supplementary Materials

The following are available online at https://www.mdpi.com/article/10.3390/cancers13215562/s1, Figure S1: Circulating HGF is correlated to higher c-Met expression by peripheral monocytes in GC patients, Figure S2: Phenotypic features of DCs generated from monocytes of gastric cancer patients in the presence of HGF, Figure S3: C-met expression on circulating monocytes is correlated to IL-10 plasmatic levels in GC patients, Table S1: Characteristics of the survival population.

## References

[1] Tanchot C., Terme M., Pere H., Tran T., Benhamouda N., Strioga M., Banissi C., Galluzzi L., Kroemer G., Tartour E. (2013). Tumor-infiltrating regulatory T cells: Phenotype, role, mechanism of expansion in situ and clinical significance. Cancer Microenviron..

[2] Terme M., Pernot S., Marcheteau E., Sandoval F., Benhamouda N., Colussi O., Dubreuil O., Carpentier A.F., Tartour E., Taieb J. (2013). VEGFA-VEGFR Pathway Blockade Inhibits Tumor-Induced Regulatory T-cell Proliferation in Colorectal Cancer. Cancer Res..

[3] Bergers G., Hanahan D. (2008). Modes of resistance to anti-angiogenic therapy. Nat. Rev. Cancer.

[4] Trusolino L., Bertotti A., Comoglio P.M. (2010). MET signalling: Principles and functions in development, organ regeneration and cancer. Nat. Rev. Mol. Cell Biol..

[5] Anestis A., Zoi I., Karamouzis M.V. (2018). Current advances of targeting HGF/c-Met pathway in gastric cancer. Ann. Transl. Med..

[6] Yu S., Yu Y., Zhao N., Cui J., Li W., Liu T. (2013). c-Met as a Prognostic Marker in Gastric Cancer: A Systematic Review and Meta-Analysis. PLoS ONE.

[7] Peng Z., Zhu Y., Wang Q., Gao J., Li Y., Li Y., Ge S., Shen L. (2014). Prognostic Significance of MET Amplification and Expression in Gastric Cancer: A Systematic Review with Meta-Analysis. PLoS ONE.

[8] Toiyama Y., Yasuda H., Saigusa S., Matushita K., Fujikawa H., Tanaka K., Mohri Y., Inoue Y., Goel A., Kusunoki M. (2012). Co-expression of hepatocyte growth factor and c-Met predicts peritoneal dissemination established by autocrine hepatocyte growth factor/c-Met signaling in gastric cancer. Int. J. Cancer.

[9] Park D.J., Yoon C., Thomas N., Ku G.Y., Janjigian Y.Y., Kelsen D.P., Ilson D.H., Goodman K.A., Tang L.H., Strong V.E. (2014). Prognostic Significance of Targetable Angiogenic and Growth Factors in Patients Undergoing Resection for Gastric and Gastroesophageal Junction Cancers. Ann. Surg. Oncol..

[10] Papaccio F., della Corte C.M., Viscardi G., di Liello R., Esposito G., Sparano F., Ciardiello F., Morgillo F. (2018). HGF/MET and the Immune System: Relevance for Cancer Immunotherapy. Int. J. Mol. Sci..

[11] Ilangumaran S., Villalobos-Hernandez A., Bobbala D., Ramanathan S. (2016). The hepatocyte growth factor (HGF)-MET receptor tyrosine kinase signaling pathway: Diverse roles in modulating immune cell functions. Cytokine.

[12] Benkhoucha M., Santiago-Raber M.-L., Schneiter G., Chofflon M., Funakoshi H., Nakamura T., Lalive P.H. (2010). Hepatocyte growth factor inhibits CNS autoimmunity by inducing tolerogenic dendritic cells and CD25+Foxp3+ regulatory T cells. Proc. Natl. Acad. Sci. USA.

[13] Benkhoucha M., Molnarfi N., Dunand-Sauthier I., Merkler D., Schneiter G., Bruscoli S., Riccardi C., Tabata Y., Funakoshi H., Nakamura T. (2014). Hepatocyte growth factor limits autoimmune neuroinflammation via glucocorticoid-induced leucine zipper expression in dendritic cells. J. Immunol..

[14] Owusu B., Galemmo R., Janetka J., Klampfer L. (2017). Hepatocyte Growth Factor, a Key Tumor-Promoting Factor in the Tumor Microenvironment. Cancers.

[15] Beppu K., Uchiyama A., Morisaki T., Matsumoto K., Nakamura T., Tanaka M., Katano M. (2001). Hepatocyte growth factor production by peripheral blood mononuclear cells of recurrent cancer patients. Anticancer Res..

[16] Malka D., François E., Penault-Llorca F., Castan F., Bouché O., Bennouna J., Ghiringhelli F., de la Fouchardière C., Borg C., Samalin E. (2019). FOLFOX alone or combined with rilotumumab or panitumumab as first-line treatment for patients with advanced gastroesophageal adenocarcinoma (PRODIGE 17-ACCORD 20-MEGA): A randomised, open-label, three-arm phase II trial. Eur. J. Cancer.

[17] Amemiya H., Kono K., Itakura J., Tang R.F., Takahashi A., An F.Q., Kamei S., Iizuka H., Fujii H., Matsumoto Y. (2002). c-Met expression in gastric cancer with liver metastasis. Oncology.

[18] Ziegler-Heitbrock L. (2015). Blood Monocytes and Their Subsets: Established Features and Open Questions. Front. Immunol..

[19] Pernot S., Voron T., Perkins G., Lagorce-Pages C., Berger A., Taieb J. (2015). Signet-ring cell carcinoma of the stomach: Impact on prognosis and specific therapeutic challenge. World J. Gastroenterol..

[20] Pernot S., Terme M., Radosevic-Robin N., Castan F., Badoual C., Marcheteau E., Penault-Llorca F., Bouche O., Bennouna J., Francois E. (2020). Infiltrating and peripheral immune cell analysis in advanced gastric cancer according to the Lauren classification and its prognostic significance. Gastric Cancer.

[21] Oku M., Okumi M., Shimizu A., Sahara H., Setoyama K., Nishimura H., Sada M., Scalea J., Ido A., Sachs D.H. (2012). Hepatocyte growth factor sustains T regulatory cells and prolongs the survival of kidney allografts in major histocompatibility complex-inbred CLAWN-miniature swine. Transplantation.

[22] Molnarfi N., Benkhoucha M., Juillard C., Bjarnadóttir K., Lalive P.H. (2014). The neurotrophic hepatocyte growth factor induces protolerogenic human dendritic cells. J. Neuroimmunol..

[23] Rutella S., Bonanno G., Procoli A., Mariotti A., de Ritis D.G., Curti A., Danese S., Pessina G., Pandolfi S., Natoni F. (2006). Hepatocyte growth factor favors monocyte differentiation into regulatory interleukin (IL)-10++IL-12low/neg accessory cells with dendritic-cell features. Blood.

[24] Molnarfi N., Benkhoucha M., Bjarnadóttir K., Juillard C., Lalive P.H. (2012). Interferon–β Induces Hepatocyte Growth Factor in Monocytes of Multiple Sclerosis Patients. PLoS ONE.

[25] Benkhoucha M., Senoner I., Lalive P.H. (2020). C-Met is expressed by highly autoreactive encephalitogenic CD8+ cells. J. Neuroinflammation.

[26] Komarowska I., Coe D., Wang G., Haas R., Mauro C., Kishore M., Cooper D., Nadkarni S., Fu H., Steinbruchel D.A. (2015). Hepatocyte Growth Factor Receptor c-Met Instructs T Cell Cardiotropism and Promotes T Cell Migration to the Heart via Autocrine Chemokine Release. Immunity.

[27] Galimi F., Cottone E., Vigna E., Arena N., Boccaccio C., Giordano S., Naldini L., Comoglio P.M. (2001). Hepatocyte growth factor is a regulator of monocyte-macrophage function. J. Immunol..

[28] Zhao L., Wu Y., Xie X.-D., Chu Y.-F., Li J.-Q., Zheng L. (2015). c-Met identifies a population of matrix metalloproteinase 9-producing monocytes in peritumoural stroma of hepatocellular carcinoma. J. Pathol..

[29] Moghul A., Lin L., Beedle A., Kanbour-Shakir A., DeFrances M.C., Liu Y., Zarnegar R. (1994). Modulation of c-MET proto-oncogene (HGF receptor) mRNA abundance by cytokines and hormones: Evidence for rapid decay of the 8 kb c-MET transcript. Oncogene.

[30] Boccaccio C., Gaudino G., Gambarotta G., Galimi F., Comoglio P.M. (1994). Hepatocyte growth factor (HGF) receptor expression is inducible and is part of the delayed-early response to HGF. J. Biol. Chem..

[31] Gayyed M.F., El-Maqsoud N.M.R.A., El-Heeny A.A.E.H., Mohammed M.F. (2015). c-MET expression in colorectal adenomas and primary carcinomas with its corresponding metastases. J. Gastrointest. Oncol..

[32] Chen P.-M., Liu K.-J., Hsu P.-J., Wei C.-F., Bai C.-H., Ho L.-J., Sytwu H.-K., Yen B.L. (2014). Induction of immunomodulatory monocytes by human mesenchymal stem cell-derived hepatocyte growth factor through ERK1/2. J. Leukoc. Biol..

[33] Mougiakakos D., Choudhury A., Lladser A., Kiessling R., Johansson C.C. (2010). Regulatory T cells in cancer. Adv. Cancer Res..

[34] Kaga T., Kawano H., Sakaguchi M., Nakazawa T., Taniyama Y., Morishita R. (2012). Hepatocyte growth factor stimulated angiogenesis without inflammation: Differential actions between hepatocyte growth factor, vascular endothelial growth factor and basic fibroblast growth factor. Vascul. Pharmacol..

[35] Zhang Y.W., Su Y., Volpert O.V., Woude G.F.V. (2003). Hepatocyte growth factor/scatter factor mediates angiogenesis through positive VEGF and negative thrombospondin 1 regulation. Proc. Natl. Acad. Sci. USA.

[36] Gerritsen M.E., Tomlinson J.E., Zlot C., Ziman M., Hwang S. (2003). Using gene expression profiling to identify the molecular basis of the synergistic actions of hepatocyte growth factor and vascular endothelial growth factor in human endothelial cells. Br. J. Pharmacol..

[37] Kopetz S., Hoff P.M., Morris J.S., Wolff R.A., Eng C., Glover K.Y., Adinin R., Overman M.J., Valero V., Wen S. (2010). Phase II trial of infusional fluorouracil, irinotecan, and bevacizumab for metastatic colorectal cancer: Efficacy and circulating angiogenic biomarkers associated with therapeutic resistance. J. Clin. Oncol..

[38] Patel M.B., Pothula S.P., Xu Z., Lee A.K., Goldstein D., Pirola R.C., Apte M.V., Wilson J.S. (2014). The role of the hepatocyte growth factor/c-MET pathway in pancreatic stellate cell-endothelial cell interactions: Anti-angiogenic implications in pancreatic cancer. Carcinogenesis.

[39] Abounader R., Lal B., Luddy C., Koe G., Davidson B., Rosen E.M., Laterra J. (2002). In vivo targeting of SF/HGF and c-met expression via U1snRNA/ribozymes inhibits glioma growth and angiogenesis and promotes apoptosis. FASEB J..

[40] Voron T., Colussi O., Marcheteau E., Pernot S., Nizard M., Pointet A.-L., Latreche S., Bergaya S., Benhamouda N., Tanchot C. (2015). VEGF-A modulates expression of inhibitory checkpoints on CD8+ T cells in tumors. J. Exp. Med..

[41] Lapeyre-Prost A., Terme M., Pernot S., Pointet A.-L., Voron T., Tartour E., Taieb J. (2017). Immunomodulatory Activity of VEGF in Cancer. Int. Rev. Cell Mol. Biol..

[42] FYakes M., Chen J., Tan J., Yamaguchi K., Shi Y., Yu P., Qian F., Chu F., Bentzien F., Cancilla B. (2011). Cabozantinib (XL184), a novel MET and VEGFR2 inhibitor, simultaneously suppresses metastasis, angiogenesis, and tumor growth. Mol. Cancer Ther..

[43] Kwilas A.R., Ardiani A., Donahue R.N., Aftab D.T., Hodge J.W. (2014). Dual effects of a targeted small-molecule inhibitor (cabozantinib) on immune-mediated killing of tumor cells and immune tumor microenvironment permissiveness when combined with a cancer vaccine. J. Transl. Med..

[44] Apolo A.B., Nadal R., Tomita Y., Davarpanah N.N., Cordes L.M., Steinberg S.M., Cao L., Parnes H.L., Costello R., Merino M.J. (2020). Cabozantinib in patients with platinum-refractory metastatic urothelial carcinoma: An open-label, single-centre, phase 2 trial. Lancet Oncol..

[45] Choueiri T.K., Powles T., Burotto M., Bourlon M.T., Zurawski B., Juárez V.M.O., Hsieh J.J., Basso U., Shah A.Y., Suarez C. (2020). 696O_PR Nivolumab + cabozantinib vs sunitinib in first-line treatment for advanced renal cell carcinoma: First results from the randomized phase III CheckMate 9ER trial. Ann. Oncol..

[46] Powles T. (2021). Recent eUpdate to the ESMO Clinical Practice Guidelines on renal cell carcinoma on cabozantinib and nivolumab for first-line clear cell renal cancer: Renal cell carcinoma: ESMO Clinical Practice Guidelines for diagnosis, treatment and follow-up 1. Ann. Oncol..

