# Supplementary material for: Targeting HGF/c-Met Axis Decreases Circulating Regulatory T Cells Accumulation in Gastric Cancer Patients
Source: Cancers (Basel). 2021 Nov 5;13(21):5562. doi: 10.3390/cancers13215562 (PMC8583551; doi:10.3390/cancers13215562)
Supplement: Supplementary file 1 [file cancers-13-05562-s001.zip › cancers-1377643-supplementary.pdf]

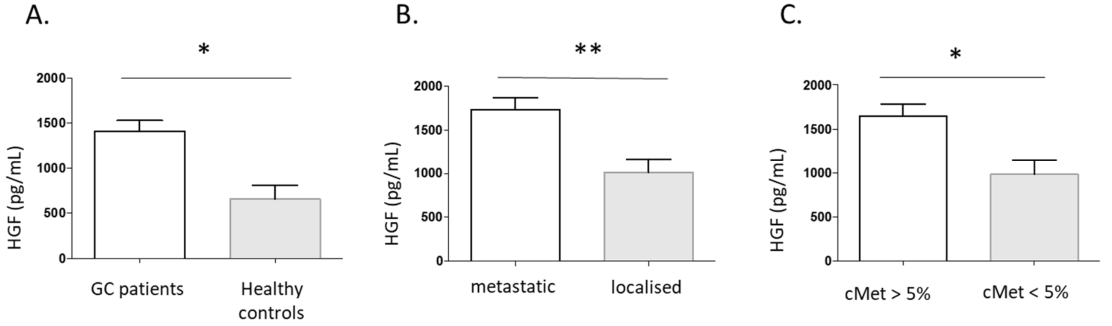

Figure S1. Circulating HGF is correlated to higher c-Met expression by peripheral monocytes in GC patients. HGF plasmatic levels were assessed by ELISA in GC patients of the HEGP cohort and in healthy controls (HC). (A) HGF plasmatic levels in GC patients (n=22; mean+/-SD: 1407+/-126pg/mL) and in HC (n=4; mean+/-SD: 658.6+/- 153pg/mL). * indicates a P value ≤ 0.05 according to the Mann Whitney test. (B) HGF plasmatic levels in GC patients with metastatic disease (n=10; mean+/-SD: 1726+/-136.3pg/mL) and with localized disease (n=6; mean+/-SD: 1010+/-152.9pg/mL). ** indicates a P value ≤ 0.01 according to the Mann-Whitney test. (C) HGF plasmatic levels in GC patients with c-Met expression by circulating monocytes >5% (n=14; mean+/-SD: 1651+/-133.2pg/mL) and in GC patients with c-Met expression by circulating monocytes<5% (n=10; mean+/-SD: 980.6+/163.6pg/mL) * indicates a P value ≤ 0.05 according to the Mann Whitney test.

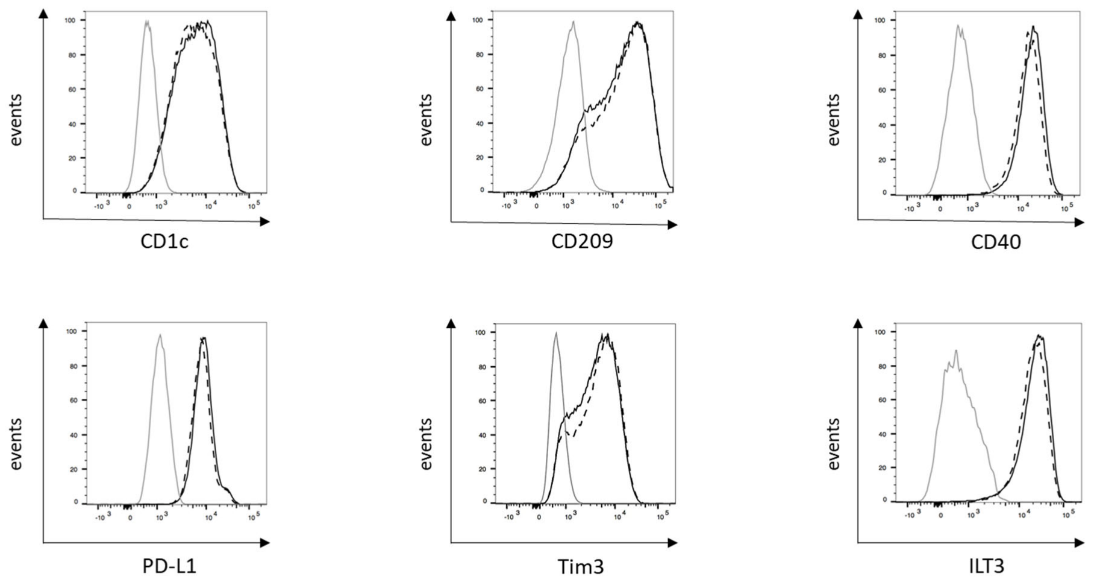

Figure S2. Phenotypic features of DCs generated from monocytes of gastric cancer patients in the presence of HGF. Freshly isolated peripheral monocytes from GC patients were cultured in the presence of GM-CSF and IL-4 with HGF (= HGF condition) or without HGF (= control condition) as described in the material and method section. After 6 days, cell phenotype was assessed by flow cytometry. Expression of CD1c, CD209, CD40, PD-L1, Tim-3 and ILT-3 in DCs generated without (black dashed curve) or with HGF (black full curve). Markers were set on their proper control isotype (light grey curve). A representative staining out of 5 to 11 independent experiments is shown.

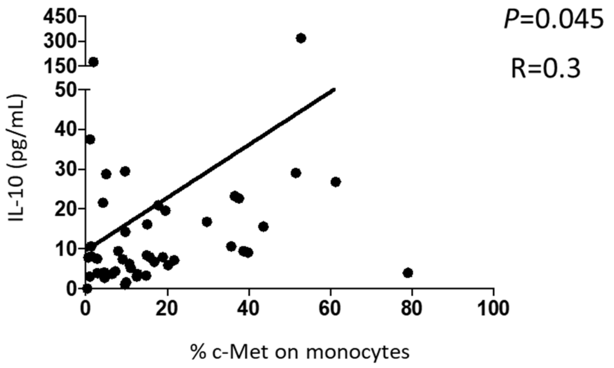

Figure S3. C-met expression on circulating monocytes is correlated to IL-10 plasmatic levels in GC patients. C-Met expression on peripheral monocytes was assessed by immunostaining as described in Fig.1. IL-10 plasmatic levels were assessed by ELISA. The correlation between IL-10 plasmatic levels and percentages of c-Met positive monocytes has been assessed for 42 patients using Spearman correlation test.

	**cMet<20% (N=21)**<br>**N (%)**	**cMet>20% (N=16)**<br>**N (%)**
**Age (years)**		
median	68	67
**Sex**		
male	11 (52)	13 (81)
female	10 (48)	43 (19)
**Localisation**		
oesogastric junction	5 (24)	6 (37)
gastric body	16 (76)	10 (63)
**Linitis**		
yes	3 (14)	1 (6)
no	14 (67)	13 (81)
mixed	4 (19)	2 (13)
**Disease status**		
localised	8 (38)	3 (19)
metastatic	13 (62)	13 (81)

Table S1. Characteristics of the survival population.
